# Identification of QTL and Qualitative Trait Loci for Agronomic Traits Using SNP Markers in the Adzuki Bean

**DOI:** 10.3389/fpls.2017.00840

**Published:** 2017-05-19

**Authors:** Yuan Li, Kai Yang, Wei Yang, Liwei Chu, Chunhai Chen, Bo Zhao, Yisong Li, Jianbo Jian, Zhichao Yin, Tianqi Wang, Ping Wan

**Affiliations:** ^1^Key Laboratory of New Technology in Agricultural Application, College of Plant Science and Technology, Beijing University of AgricultureBeijing, China; ^2^Beijing Genomics Institute-ShenzhenShenzhen, China; ^3^College of Plant Science, Jilin UniversityChangchun, China

**Keywords:** adzuki bean (*Vigna angularis*), agronomic trait, QTL, qualitative trait, SNP marker, candidate gene

## Abstract

The adzuki bean (*Vigna angularis*) is an important grain legume. Fine mapping of quantitative trait loci (QTL) and qualitative trait genes plays an important role in gene cloning, molecular-marker-assisted selection (MAS), and trait improvement. However, the genetic control of agronomic traits in the adzuki bean remains poorly understood. Single-nucleotide polymorphisms (SNPs) are invaluable in the construction of high-density genetic maps. We mapped 26 agronomic QTLs and five qualitative trait genes related to pigmentation using 1,571 polymorphic SNP markers from the adzuki bean genome via restriction-site-associated DNA sequencing of 150 members of an F_2_ population derived from a cross between cultivated and wild adzuki beans. We mapped 11 QTLs for flowering time and pod maturity on chromosomes 4, 7, and 10. Six 100-seed weight (SD100WT) QTLs were detected. Two major flowering time QTLs were located on chromosome 4, firstly *VaFld4.1* (PEVs 71.3%), co-segregating with SNP marker s690-144110, and *VaFld4.2* (PEVs 67.6%) at a 0.974 cM genetic distance from the SNP marker s165-116310. Three QTLs for seed number per pod (*Snp3.1, Snp3.2*, and *Snp4.1*) were mapped on chromosomes 3 and 4. One QTL *VaSdt4.1* of seed thickness (SDT) and three QTLs for branch number on the main stem were detected on chromosome 4. QTLs for maximum leaf width (LFMW) and stem internode length were mapped to chromosomes 2 and 9, respectively. Trait genes controlling the color of the seed coat, pod, stem and flower were mapped to chromosomes 3 and 1. Three candidate genes, *VaAGL, VaPhyE*, and *VaAP2*, were identified for flowering time and pod maturity. *VaAGL* encodes an agamous-like MADS-box protein of 379 amino acids. *VaPhyE* encodes a phytochrome E protein of 1,121 amino acids. Four phytochrome genes (*VaPhyA1, VaPhyA2, VaPhyB*, and *VaPhyE*) were identified in the adzuki bean genome. We found candidate genes *VaAP2/ERF.81* and *VaAP2/ERF.82* of SD100WT, *VaAP2-s4* of SDT, and *VaAP2/ERF.86* of LFMW. A candidate gene *VaUGT* related to black seed coat color was identified. These mapped QTL and qualitative trait genes provide information helpful for future adzuki bean candidate gene cloning and MAS breeding to improve cultivars with desirable growth periods, yields, and seed coat color types.

## Introduction

The adzuki bean (*Vigna angularis*) is an important diploid pulse crop (2n = 2x = 22) that is rich in easily digestible protein with extremely low fat content (Lin, 2002). It was domesticated about 12,000 years ago in China (Liu et al., 2013), and is cultivated today in over 30 countries of the world, principally those of eastern and northern Asia (Tomooka et al., 2002; Kramer et al., 2012). China is the largest producer of adzuki beans in the world, with an area of approximately 25,000 ha cultivated annually (Cheng and Tian, 2009). The adzuki bean is a rich source of phenolic compounds, flavonoids, vitamin A, vitamin B, iron, zinc, and folate (Amarowicz et al., 2008; Yao et al., 2012).

Gene and QTL mapping is very important for gene cloning, MAS breeding, and trait improvement; however, only a few studies have focused on mapping the QTL and the qualitative trait genes in the adzuki bean. The QTLs of 21 domestication-related traits were first mapped to different linkage groups by 21 polymorphic SSR markers using the same BC_1_F_1_,F_2_,and F_2:3_ populations to construct a molecular linkage map (Han et al., 2005). Most traits mapped to particular regions of linkage groups (LGs) 1, 2, 4, 7, and 9. Pod size, germination efficacy, seed size, and lower stem length mapped to LGs 1 and 2. The QTLs of LGs 7 and 9 were associated with upper-stem length, maximum leaf size, and pod and seed sizes (Isemura et al., 2007).

Kaga et al. (2008) used 316 SSR primer pairs from the adzuki bean (Wang et al., 2004), 170 SSR primer pairs from the common bean, 45 cowpea SSR primer pairs, and AFLPs to screen for polymorphisms in the two parents. In total, 176 adzuki bean SSRs and 5 common bean SSR primer pairs exhibited clear polymorphisms. F_2_ mapping population consisted of 188 plants derived from crosses between the Japanese wild bean (*V. angularis* var. *nipponensis*) and the cultivated adzuki bean (*V. angularis var. angularis*). The AFLP approach was developed to fill a large gap (~40 cM) in the center of LG9. In total, 233 markers (191 SSRs, 2 STSs, 1 CAPS, 2 SCARs, and 36 AFLPs) and three morphological traits were mapped to 10 linkage groups (one less than the 11 haploid chromosomes of the adzuki bean). One linkage group, termed “LG4+6,” contained the LG4 and LG6 markers of previous maps. In total, 162 QTLs influencing 46 domestication-related traits were identified. The QTLs affecting seed dormancy; seedling stem length; red seed-coat color; and the organ sizes of seeds, pods, and leaves, were mapped to LG1. The QTLs for pod dehiscence, length, size, and color lay on LG7. The QTLs for organ size, growth habit, and yield-related traits (total seed weight, total pod and seed numbers, 100-seed weight, and total seed weight), maximum leaflet length, primary leaf width, and pod width and length lay in two distinct regions of LG9 (Kaga et al., 2008).

*Azuki Dwarf1* (AD1), a single genetically unstable dwarf locus, co-segregates with SSRs on LG4 (Aoyama et al., 2011). A strong QTL for seed coat color, designated *OLB1*, explains 54.43 and 56% of the total variances in the L^*^ (lightness), a^*^ (redness), and b* (yellowness) values. In addition, a minor QTL, designated *OLB2*, explains 6% of the total variance in redness. *OLB1* and *OLB2* are located in LG1. Furthermore, two traits controlled by a single Mendelian gene: *IVY* (ivory/yellow) and *POB* (pale olive/buff) (seed coat colors) are located in LG8 and LG10, respectively (Horiuchi et al., 2015).

We previously published a draft version of the adzuki bean genome (Yang et al., 2015), which will facilitate the identification of agronomic trait genes and accelerate the improvement of adzuki bean.

However, to the best of our knowledge, no QTL analysis using high-density segregated SNPs has been performed in adzuki bean. In this study, we initially collected phenotypic data and then defined genotypic data using SNP markers via restriction-site-associated DNA (RAD) sequencing. The QTLs of important agronomic traits of the adzuki bean were mapped using these polymorphic SNP markers. Our results elucidate how genetic features control the agronomic traits of the adzuki bean, and the major QTLs and genes that we have identified will expedite MAS breeding and the improvement of these traits in adzuki bean.

## Materials and methods

### The mapping population

The F_2_ mapping population initially comprised 250 individuals derived by crosses between an adzuki bean cultivar (Ass001) and a wild adzuki bean (accession # CWA108) collected in China. The F_2_ population, and 10 plants of each parent, were grown in the Experimental Farm of Beijing University of Agriculture (BUA) from June to October, 2013. A single seed was planted with 60-cm row spacing and 30-cm plant spacing. Each F_2:3_ line had two rows that were 3 m long and 45 cm wide; 35 seeds were evenly planted in each row in the field in June 2014 at the BUA Experimental Farm. From the center of the rows, 10 representational plants were selected to evaluate traits, and 10 plants from each parent were grown together with the F_2:3_ line. The RIL (Recombinant Inbred lines) of F_3_ were obtained from single seed descendent of the F_2_ individuals; their parents were grown in the same manner as F_2_ at the BUA Experimental Farm in 2014 and 2015. F_3:4_ lines and parents were planted and evaluated for traits in the same manner as F_2:3_ lines at the BUA Experimental Farm in 2015.

We selected 150 typical F_2_ individuals to extract DNA for RAD sequencing.

### Trait measurements

In total, 28 traits, including 24 quantitative, and 4 qualitative traits, were evaluated in F_2_, F_3_, and F_4_ generations from 2013 to 2015 (Table 1; Table S1). Morphology was investigated according to a published standard (Tomooka et al., 2002; Cheng et al., 2012). The traits included the color of the seed-coat, pod, stem and flower; the first-to-tenth internodal length; the maximal leaf length and width; the maximum leaflet area; and the growth habit at 50% of flowering; we investigated plant height, stem diameter, the number of branches, flowering time, and pod maturing time; and 100-seed weight, seed size, and pod size were measured after harvesting, respectively. The maximal leaf lengths and widths, and leaflet areas, were estimated with the aid of a YMJ-C leaf area meter (Zhejiang Top Instrument Co., Ltd., China). The lengths, widths, and thicknesses of 10 seeds were measured using digital calipers.

**Table 1:** **Traits examined and the evaluation method**.

**Trait**	**Trait abbreviation**	**Evaluation method**
Plant height (cm)	PHT	Plant height from cotyledonary node to tip of mature plant
Stem thickness (cm)	STT	Stem diameter below the primary leaf
Branch position of first branch	BRP	Position of 1st branch on main stem from 1st trifoliate leaf node to 10th trifoliate leaf node
Branch number on main stem	BRN	Number of branches on main stem from 1st trifoliate leaf node to 10th trifoliate leaf node
Stem internode length (first to tenth) (cm)	ST1I–ST10I	Length from primary leaf node to each node
Pod length (cm)	PDL	Length of straight pod
Pod width (cm)	PDW	Width of pod at widest part
Pod number per plant	PDTN	Pod number per plant (10 plants)
Seed number per pod	SDNPPD	Seed number per pod (10 pods)
Total seed number.	SDTN	Seed number per plant (10 plants)
Total seed weight	SDTWT	Seed weight per plant (10 plants)
100 seed weight (g)	SD100WT	Weight of 100 seeds
Seed length (mm)	SDL	Maximum distance from top to bottom of the seed
Seed width (mm)	SDW	Maximum distance from hilum to its opposite side
Seed thickness (mm)	SDT	Maximum distance between the two sides of the hilum
Pod color	PDC	Black, straw
Seed coat color	SDC	Black, brown, red
Growth habit	GH	Stem twining beyond the main stem 10th internode (erect, semi-erect, twining)
Stem color	STC	Purple, green
Days to first flower (days)	FLD	Number of days from sowing to first flowering
Days to 50% flower (days)	FLD50	Number of days from sowing to 50% flowering
Days to maturity of 25% pods (days)	PDDM25	Number of days from sowing to 25% mature pods
Days to maturity of 50% pods (days)	PDDM50	Number of days from sowing to 50% mature pods
Days to maturity of 75% pods (days)	PDDM75	Number of days from sowing to 75% mature pods
Days to maturity of 100% pods (days)	PDDM100	Number of days from sowing to 100% mature pods
Maximum leaf area (cm^2^)	LFMA	Leaflet area of the largest terminal leaflet on a leaf between 1st trifoliate leaf node and 10th trifoliate leaf node
Maximum leaf length (cm)	LFML	Length of largest terminal leaflet on a leaf between 1st trifoliate leaf node and 10th trifoliate leaf node
Maximum leaf width (cm)	LFMW	Width of largest terminal leaflet on a leaf between 1st trifoliate leaf node and 10th trifoliate leaf node

### RAD-sequencing and SNP detection

Genomic DNA of 150 F_2_ individual plants and 10 plants of their parents were selected from which to extract genomic DNA from young leaves using the CTAB protocol. DNA was digested with *EcoRI* using the method of Baird et al. (2008) with minor modifications for RAD sequencing. SNP detection and genetic map construction followed the method of Yang et al. (2015).

### Data analysis and QTL mapping

We calculated the mean, the standard error of the mean, and broad-sense heritability (the ${\text{H}}_{\text{B}}^{2}$ value) of investigated 24 quantitative traits, including plant height, organ size, yield, flowering time and maturing period, and plant architecture (Table 1) for parents, and the F_2_, F_3_, F_4_ populations. The heritability was computed using a method of regression for progeny values on parental values. The calculation of ${\text{H}}_{\text{B}}^{2}$ employed the formula: ${\text{H}}_{\text{B}}^{2}$ = *b*_*F*2·*F*2:3_ = *COV*_*F*2·*F*2:3_*/V*_*F*2_.

Using RAD tag technology, SNPs were identified among 150 F_2_ individuals derived from a cross of cultivar Ass001 (P1) with the wild adzuki bean accession no. CWA108 (P2). After genotyping, a linkage map was created using *JoinMap 4.0* software (Van Ooijen, 2006) running F_2_ population-type codes. Markers exhibiting distorted segregation (*p* < 0.01, Chi-squared test) were excluded (Grattapaglia and Sederoff, 1994). The remaining 1,571 markers were used to construct a genetic map. Eleven linkage groups (for adzuki bean, 2n = 22 chromosomes) were formed with the logarithm-of-the-odds (LOD) score set to 6.0 (Yang et al., 2015) and ordered using a regression mapping algorithm. Recombination frequencies were translated to genetic distances using Kosambi's mapping function (Kosambi, 1994). Qualitative trait genes were mapped using *JoinMap 4*.*1*.

Five Mendelian phenotype markers (stem color, flower color, pod color, and two seed coat colors) were detected as described in the Methods section. These phenotype markers were used as molecular markers for genotyping and linkage grouping.

Further QTL analysis was performed using MapQTL 6.0 software, which is widely used in the analysis of QTLs (Van Ooijen, 2009). First, the PERMUTATION test was used to obtain genome-wide LOD thresholds (*p* < 0.05), and each trait was subjected to 10,000 permutations to derive the empirical LOD threshold (Churchill and Doerge, 1994). Next, the regression approach of the interval mapping model was introduced to obtain LOD values for all the significant markers, and these were associated with candidate traits. All mapping markers for which the LOD value was equal to or greater than the LOD threshold value were retained. Finally, these significant markers were used as cofactors in the multiple QTL method (MQM) (Jansen, 1993), such that the identified markers were adjacent to the significant QTLs in each group. All mapping information including chromosomal location, magnitude, direction of the additive effect, and the proportion of the phenotypic variation explained (PVE) in each detected QTL was obtained from the MQM outputs. Markers with LODs greater than the LOD threshold were identified and were regarded as the optimal final markers.

### Candidate gene identification and phylogenetic analysis

Based on candidate gene sequences of *E3*, which encodes phyA-type photoreceptors involved in the control of flowering and maturity in soybean (Liu et al., 2008; Watanabe et al., 2009), transcription factor agamous-like MADS-box and *AP2* were detected in the mapping regions for flowering time, maturity, and seed coat color. We downloaded 427 AP2 gene sequences, 123 MADS gene sequences, 95 *PHY* gene sequences, and 150 UGT gene (UDP flavonoid glycosyl transferase) sequences of *Arabidopsis thaliana* from NCBI. All of these protein sequences were used as seed sequences during gene copy number analysis. These four gene types were searched for and identified from the adzuki bean genome (PRJNA261643), and other sequenced legume genomes like soybean (phytozomev10), chickpea (http://gigadb.org /dataset/100076), *Medicago truncatula* (phytozomev10), pigeonpea (http://gigadb.org/dataset/100028), common bean (phytozomev10), mung bean (ftp.ncbi.nih.gov) and *Arachis duranensis* (http://www.peanutbase.org/), and from the genomes of *Arabidopsis* (TAIR9, Phytozome v10.0), *Brassica rapa* (Phytozome v10.0), rice (http://rice.plantbiology.msu.edu/), maize (phytozomev10). Previously published related gene sequences from *Arabidopsis* genomes were collected and used as query sequences. These query sequences were then used to align each sequenced legume genome sequence using TBLASTN v2.2.23 with a threshold *E*-value less than 10^−10^. Because we obtained so many alignment results within the nearby genomic area, we extracted high quality alignments (query_align_ratio, the ratio of alignment length of query sequences size ≥ 70% and identity ≥ 40%). Functional intact genes were confirmed via collection of blast-hits using the above method. Each of the blast-hit sequences was then extended in both 3′ and 5′ directions along the genome sequences to predict gene structure using Genewise. The resulting sequences were further confirmed by phylogenetic structure analysis. Finally, the coding sequences with proper ATG or the stop codon were extracted, but not those with interrupting stop codons or frame shifts.

## Results

### Phenotypic variation and genetic analysis

**Figure 1:**
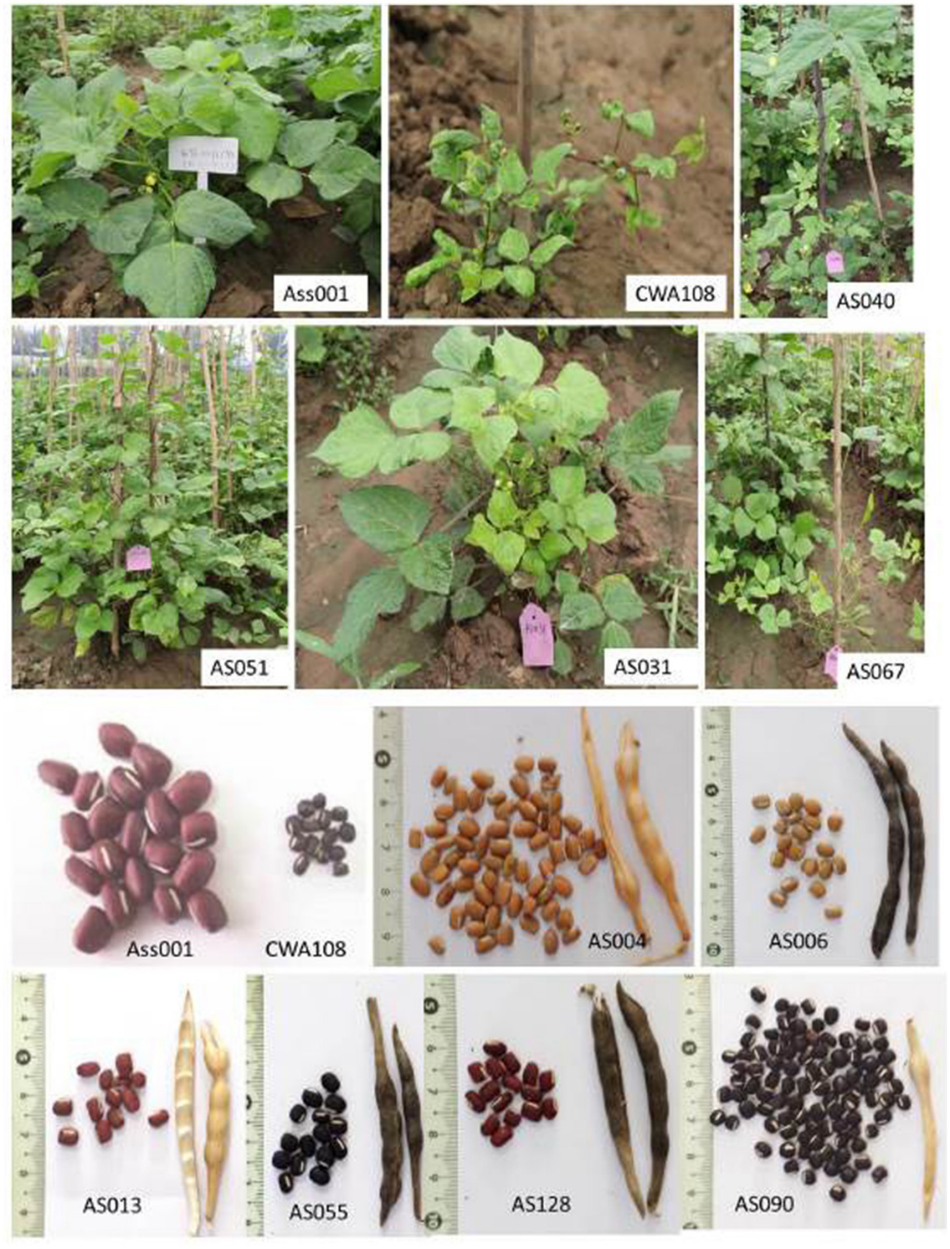
**Phenotype of parents (Ass001 and CWA108) and several individuals in the F_**2**_ mapping population Ass001: female parent; CWA108: male parent; AS: individuals of F_**2**_**.

The phenotypes of the cultivated and wild plants differed significantly. The female parent Ass001 (a cultivar) had a red seed coat, a straw-colored pod, a green stem, an erect plant, and a large seed. The wild parent had a black seed coat, a black pod, a purple stem, a twining plant, and a small seed. The different individuals in the same generation exhibited wide segregation for agronomic traits or variation (Table S1, Table 2, Figure 1).

Table 2**The mean, standard error of mean, and heritability values for parents, the F_**2**_, F_**3**_, F_**4**_ populations of the cross between cultivated and wild adzuki bean**.

**Table:** 

	**F**_**2**_ **population**						
	**Ass001**		**CWA108**		**F**_**2**_		
	**Mean**	**SEM**	**Mean**	**SEM**	**Mean**	**SEM**	**CV**
PHT	50.60	1.81	67.80	5.93	88.70	3.20	42.66
STT	0.57	0.02	0.55	0.01	0.35	0.01	39.35
BRP	1.44	0.24	1.40	0.25	1.99	0.11	64.59
BRN	5.60	0.58	10.90	0.38	6.33	0.19	34.71
ST1I–ST10I	12.05	0.48	16.70	1.55	18.43	0.59	38.01
PDL	7.70	0.17	3.57	0.19	5.50	0.08	17.94
PDW	0.65	0.01	0.29	0.01	0.48	0.00	11.31
PDTN	25.30	3.56	20.56	1.89	26.38	2.08	93.38
SDNPPD	4.84	0.21	2.67	0.44	4.18	0.10	28.58
SDTN	123.20	18.67	54.22	8.84	118.18	10.19	102.04
SDTWT	17.11	2.54	0.90	0.13	8.34	0.75	106.13
SD100WT	13.95	0.30	1.69	0.12	6.77	0.14	24.00
SDL	0.70	0.01	0.36	0.01	0.52	0.00	10.27
SDW	0.53	0.01	0.27	0.01	0.44	0.00	8.14
SDT	0.56	0.00	0.35	0.00	0.41	0.00	7.81
FLD	57.20	1.53	81.30	0.30	77.96	1.31	19.93
FLD50	67.40	1.42	87.70	0.26	89.10	1.28	16.99
PDDM25	91.70	0.60	112.80	0.68	103.32	1.24	13.94
PDDM50	97.20	0.94	120.80	1.01	111.33	1.25	12.92
PDDM75	105.20	1.04	122.50	0.98	117.85	1.19	11.14
PDDM100	114.10	0.89	138.20	2.15	126.13	1.16	10.39
LFMA	41.47	1.54	5.72	0.50	23.45	0.67	33.49
LFML	10.02	0.22	4.28	0.18	7.61	0.13	19.39
LFMW	6.87	0.14	2.76	0.14	5.26	0.09	19.56
PDC	Straw		Black				
SDC	Red		Black				
GH	Erect		Twining				
STC	Green		Purple

**Table:** 

	**F**_3_ **population**							**Heritability F**_3_ **on F**_2_
	**Ass001**		**CWA108**		**F**_3_			
	**Mean**	**SEM**	**Mean**	**SEM**	**Mean**	**SEM**	**CV**	
PHT	42.20	2.18	64.20	8.51	73.96	3.89	54.41	24.98
STT	0.55	0.03	0.56	0.02	0.38	0.05	27.47	34.83
BRP	2.80	0.44	2.00	0.45	2.15	0.14	67.57	0.00
BRN	4.00	0.49	11.40	0.51	5.30	0.28	54.45	36.02
ST1I–ST10I	15.80	0.53	27.40	1.44	17.83	0.53	30.87	32.04
PDL	8.14	0.06	4.46	0.38	5.80	0.09	15.16	16.77
PDW	0.62	0.01	0.24	0.01	0.46	0.00	9.39	1.64
PDTN	13.90	1.93	19.60	2.38	19.99	1.92	99.35	6.80
SDNPPD	5.19	0.13	5.00	0.63	5.06	0.14	28.21	6.95
SDTN	72.90	7.51	95.00	12.03	111.20	11.86	110.30	12.72
SDTWT	12.70	1.07	1.33	0.17	7.25	0.99	140.50	7.79
SD100WT	17.51	0.85	1.36	0.04	5.78	0.18	32.95	45.70
SDL	0.76	0.01	0.41	0.01	0.64	0.05	74.56	50.81
SDW	0.63	0.01	0.28	0.01	0.05	0.02	453.79	35.44
SDT	0.58	0.01	0.26	0.01	0.39	0.00	12.26	32.48
FLD	54.80	0.49	92.00	0.71	66.49	0.94	14.55	34.46
FLD50	62.20	0.49	99.00	0.95	77.75	0.85	11.35	21.96
PDDM25	76.00	0.54	128.00	0.84	82.60	0.97	9.79	18.32
PDDM50	90.00	0.56			91.80	0.78	8.31	13.62
PDDM75	100.00	0.65	135.20	1.24	99.86	1.00	8.42	13.39
PDDM100	106.00	0.79	140.00	2.49	105.78	0.95	9.32	9.75
LFMA	42.72	1.14	6.20	0.80	23.71	0.73	31.96	89.20
LFML	10.35	0.17	4.48	0.30	7.64	0.15	19.69	97.08
LFMW	7.06	0.13	2.93	0.22	5.23	0.10	20.02	98.39
PDC	Straw		Black					
SDC	Red		Black					
GH	Erect		Twining					
STC	Green		Purple					
**F**_4_ **population**								**Heritability F**_4_ **on F**_3_
	**Ass001**		**CWA108**		**F**_4_		
	**Mean**	**SEM**	**Mean**	**SEM**	**Mean**	**SEM**	**CV**	
PHT	43.75	1.42	50.20	7.14	36.40	1.40	40.05	3.68
STT	0.63	0.04	0.45	0.01	0.42	0.01	28.04	31.68
BRP	2.00	0.32	2.00	0.32	2.46	0.14	60.41	0.00
BRN	3.80	0.73	5.20	0.58	3.20	0.22	70.48	31.76
ST1I–ST10I	15.80	1.28	20.00	1.30	16.39	0.55	34.71	29.51
PDL	10.43	0.20	3.92	0.35	6.86	0.16	23.55	91.78
PDW	0.80	0.01	0.24	0.01	0.50	0.01	25.65	0.01
PDTN	17.20	1.98	16.00	2.19	21.10	1.77	86.67	35.09
SDNPPD	7.42	0.37	4.60	0.40	6.75	0.19	29.38	48.58
SDTN	90.20	9.85	88.00	6.00	104.92	10.15	99.57	33.09
SDTWT	18.82	2.56	1.63	0.22	8.14	1.00	126.76	57.17
SD100WT	20.91	0.51	2.25	0.17	6.89	0.23	34.65	73.59
SDL	0.83	0.01	0.40	0.01	0.52	0.01	12.88	52.32
SDW	0.59	0.02	0.30	0.01	0.39	0.00	12.07	53.83
SDT	0.65	0.01	0.36	0.00	0.41	0.01	15.67	55.15
FLD	50.00	1.52	95.00	0.32	54.38	0.91	17.74	43.72
FLD50	59.00	1.36	104.00	0.35	65.99	1.12	17.84	47.67
PDDM25	105.00	0.89	130.00	0.95	103.12	0.65	6.63	31.53
PDDM50								
PDDM75								
PDDM100	115.00	1.05	149.00	1.14	113.48	0.64	5.96	0.47
LFMA			6.13	0.56	24.35	0.73	31.63	94.03
LFML			4.13	0.15	7.85	0.14	18.22	93.31
LFMW			2.67	0.15	5.34	0.09	18.52	91.38
PDC	Straw		Black					
SDC	Red		Black					
GH	Erect		Twining					
STC	Green		Purple					

*Trait abbreviations are shown in Table 1. Populations of trait value was listed. SEM, standard errors of mean values. CV, coefficient of variation*.

We calculated the trait means, the standard error of the mean, and the heritability of the parental F_2_, F_3_, and F_4_ populations (Table 2). The leaf size, seed and pod sizes, seed number per pod (SDNPPD), seed total number (SDTN), and seed total weight (SDTWT) of the cultivated parent were greater than those of the wild parent. The mean values of the wild parent were greater than those of the cultivated parent for plant height, branch number on the main stem, stem internode length (from the first to the tenth internode [ST1I-ST10I]), and flowering and maturation times. The individuals in F_2_ showed wide segregation, and different lines in F_3_ or F_4_ exhibited similar phenotypic variation (Table 2). The means of various parameters of the F_2_, F_3_, and F_4_ populations generally lay between those of the cultivated and wild parents, except for plant height, ST1I-ST10I, the total number of pods per plant, days of ≥50% flowering (F_2_), and plant height, total number of pods per plant, and SDTN (F_3_). Many traits were highly heritable (>70%).

Seed coat color, pod color, and stem color are all qualitative traits. The segregation ratios of the pod color and stem color were as expected (3:1) based on the Chi-squared test. The seed coat colors of the F_2_ population included black, light brown, and red. The Chi-squared test showed that the segregation ratio of black: light brown: red seed coats was consistent with a 12:3:1 ratio; these seed coat colors were controlled by two genes. Black to light brown showed dominant epistasis (12:3, χ^2^ = 3.820 < $\chi_{0.05}^{2}$ = 3.84), light brown was dominant to red (3:1, χ^2^ = 0.701 < $\chi_{0.05}^{2}$ = 3.84).

### QTL detection and analysis

**Figure 2:**
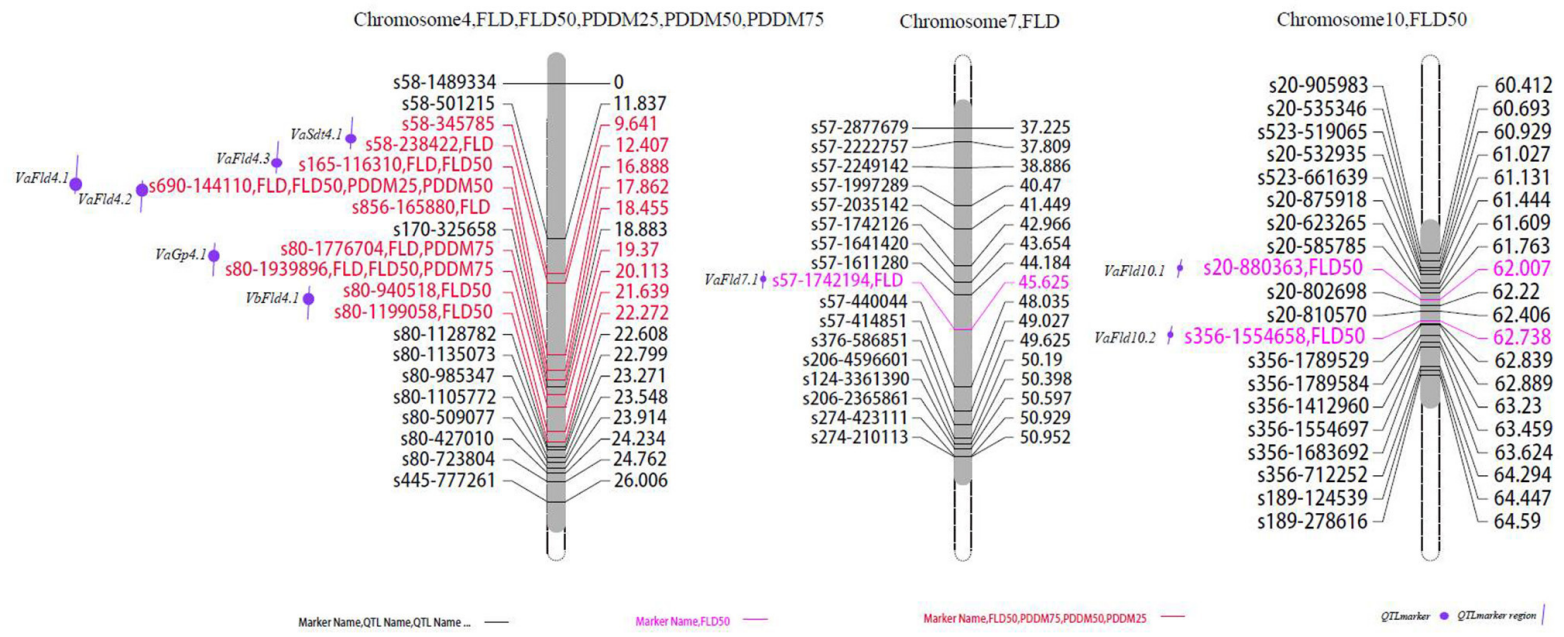
**Locations of identified QTLs for flowering and maturation in the map of the population derived from the cross between cultivated and wild adzuki bean**. FLD, days to first flowering; FLD50, days to 50% flowering; PDDM25, 25% pod maturation; PDDM50, 50% pod maturation; PDDM75, 75% pod maturation.

**Figure 3:**
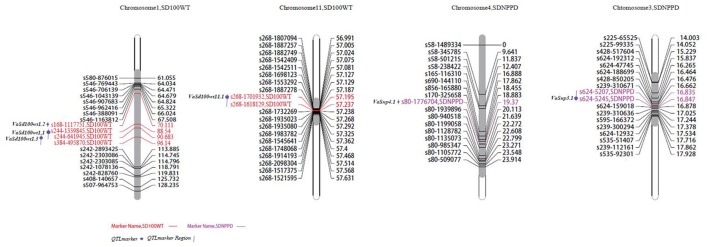
**Locations of identified QTLs for yield traits in the map of the population derived from the cross between cultivated and wild adzuki bean**. SD100WT, 100 seed weight; SDNPPD, seed number per pod.

**Figure 4:**
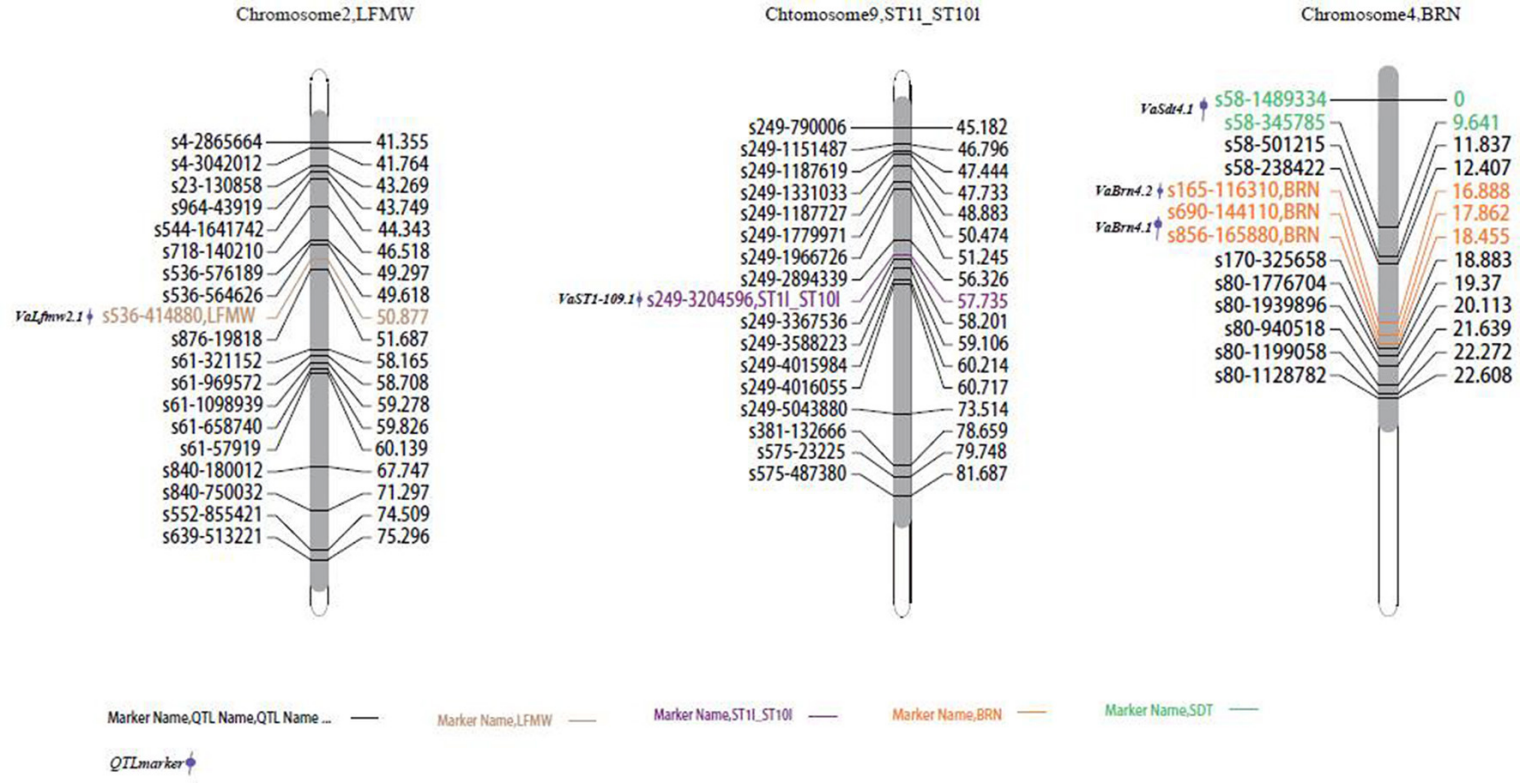
**Locations of identified QTLs for morphological traits in the map of the population derived from a cross between cultivated and wild adzuki bean**. LFMW, maximum leaf width; ST1I-ST10I, stem internode length from the first to the tenth node; BRN, branch number on the main stem; SDT, seed thickness.

We previously constructed a high-density SNP genetic map that we used only in genome assembly to anchor the scaffolds of the adzuki bean genome to the chromosomes (Yang et al., 2015); however, no morphological characteristics or QTL were involved in that SNP genetic map. The SNP genetic map was composed of 1,571 SNPs covering 11 linkage groups, spanning 1,031.17 cM, with an average of 4.33 mapped SNPs per scaffold at a mean marker distance of 0.67 cM (Table S2, Table 3). We used 1,571 polymorphic SNP markers to map QTL and qualitative trait genes in this study. In total, we identified 26 QTLs for flowering time, growth period, agronomic traits, and yield traits (Figures 2–**4** and Table 4).

**Table 3:** **Summary of the high-density SNP genetic map of adzuki beans**.

**Linkage group ID**	**Total SNP number**	**Total distance (cM)**	**Anchored scaffold length (bp)**	**Mbp/cM**
Chr 1	64	128.24	42,245,285	0.33
Chr 2	135	108.42	35,639,406	0.33
Chr 3	172	108.30	41,220,908	0.38
Chr 4	108	104.42	34,681,404	0.33
Chr 5	194	101.70	30,704,717	0.30
Chr 6	135	98.59	19,363,203	0.20
Chr 7	164	95.13	39,418,547	0.41
Chr 8	97	85.72	30,803,815	0.36
Chr 9	111	84.48	31,756,850	0.38
Chr 10	255	70.45	40,442,837	0.57
Chr 11	136	45.72	26,600,026	0.58
Total	1,571	1031.17	372,876,998	–

**Table 4:** **QTLs mapped of agronomic traits in F_**2**_, F_**3**_, and F_4_ populations**.

**Trait^a^**	**QTL**	**Chr**	**QTL peak (cM)**	**Flanking marker^b^**	**SNP (P1/P2)^c^**	**Genetic distance between loci and trait cM**	**Left flanking marker (cM)**	**Right flanking marker (cM)**	**Genomic position^d^**	**LOD**	**Expl%**
BRN	*VaBrn4.1* (F_2_,F_3_)	4	18.455	s690-144110,s856-165880	(T/G)	0.0593	17.862	18.455	2728770–2973972	5.91	20.4
	*VaBrn4.2* (F_3_)	4	17.862	s165-116310	(G/A)	0.974	16.888	16.888	2466844	4.55	20.8
ST1l_ST10l	*VaST1-10I9.1* (F_2_)	9	57.735	s249-3204596	(C/A)	0	57.735	57.735	28712717	4.92	17.3
SDNPPD	*VaSnp3.1* (F_2_)	3	16.847	s624-5207,s624-5245	(A/C)	0.0120	16.835	16.847	5754076–5754114	4.89	17.3
	*VaSnp4.1* (F_2_)	4	19.37	s80-1776704	(G/A)	0	19.37	19.37	5393066	3.98	11.8
SD100WT	*VaSd100wt11.1* (F_2_)	11	57.237	s268-1701932,s268-1618129	(C/T)	0.0420	57.195	57.237	31820598–31904338	6.00	20.7
	*VaSd100wt1.1* (F_2_,F_3_)	1	92.683	s244-1339845,s244-641945	(A/G)	2.143,13.457	90.54	106.14	32224913–32922813	4.34	12.2
	*VaSd100wt1.2* (F_3_)	1	88.54	s168-1117751	(G/A)	19.429	88.54	88.54	30134755	4.16	19.2
	*VaSd100wt1.3(F2)*	1	90.683	s244-641945,s384-495870	(G/A)	0.5457	90.683	96.14	32922813–36773639	4.06	11.5
PDDM25	*VaFld4.1* (F_2_)	4	17.862	s690-144110	(A/C)	0	17.862	17.862	2728770	20.00	55.4
PDDM50	*VaFld4.1* (F_2_)	4	17.862	s690-144110	(A/C)	0	17.862	17.862	2728770	25.5	64.9
PDDM75	*VaGp4.1* (F_2_,F_3_)	4	20.113	s80-1776704,s80-1939896	(C/T)	0.7431	19.37	21.113	5556258	14.46	47
FLD	*VaFld4.1* (F_2_/F_4_,F_2_)	4	17.862	s165-116310,s690-144110	(A/C)	0.9740	16.888	17.862	2466844–2728770	32.29	71.3
	*VaFld4.2* (F_2_,F_2_)	4	17.862	s690-144110,s856-165880	(A/C)	0.0593	17.862	18.455	2728770–2973972	32.29	25.9
	*VaFld4.3(F_4_,F_4_)*	4	16.888	s58-238422,s165-116310	(G/A)	5.4490	12.407	16.888	238422–2466844	6.19	30.3
	*VaFld7.1* (F_2_)	7	45.625	s57-1742194	(G/T)	0	45.625	45.462	8738088	4.00	4.1
FLD50	*VaFld4.1* (F_2_,F_2_)	4	17.862	s165-116310, s690-144110	(A/C)	0	16.888	17.862	2466844–2728770	33.96	73.1
	*VbFld4.1* (F_3_,F_4_)	4	22.272	s80-940518,s80-1199058	(C/A)	0.6330	21.639	22.272	4556880–4815420	4.69	24.2
	*VaFld10.1* (F_2_)	10	62.007	s20-880363, s356-1554658	(A/C)	0	62.007	62.007	35805580–37408870	4.05	3.9
	*VaFld10.2(F_2_)*	10	62.738	s356-1554658	(T/G)	0	62.735	62.735	37408870	4.00	3.9
LFMW	*VaLfmw2.1* (F_4_)	2	50.877	s536-414880	(C/G)	0	50.877	50.877	22751890	4.1	21.2
SDT	*VaSdt4.1* (F_2_)	4	9.641	s58-1489334,s58-345785	(G/T)	9.6410	0	9.641	345785,1489334	2.84	9.11

*The Permutation Test is used to calculate the significant threshold for subsequent Interval mapping and MQM mapping; “Expl(%)” denotes the percentage of the variance (Van Ooijen, 2009); “QTL Peak” presents a loci with the largest LOD value. This loci identifies the most likely location that is located within a gene or near a gene affected by a relative trait*.

a *QLT names: BRN, number of branches on the main stem; ST1l_ST10l, stem Internode length (first to tenth); SDNPPD, seed number per Pod; SD100WT, 100-seed weight of pod; PDDM25, days to maturity of 25% pod; PDDM50, days to maturity of 50% pod; PDDM75, days to maturity of 75% pod; FLD, days to first flowering; FLD50, days of 50% flowering*.

b *The left flanking marker, the right flanking marker: if there is only one marker at a site, only one marker met our criterion (LOD > threshold value of the Permutation Test)*.

c *P1 is the abbreviation of parent 1 which is one of the parents of the QLT group, P2 represents another parent*.

d *Genomic position: genomic position of the left flanking marker, genomic position of the right flanking marker*.

### Flowering time and growth period

We found 11 QTLs affecting flowering time and pod maturity on chromosomes 4, 7, and 10 (Figure 2). Most flowering and maturation time genes mapped to chromosome 4, except for the two minor QTLs *Fld4.5* and *Fld4.6*. The “days to first flowering” trait was controlled by only a few genes. Two major QTLs (*VaFld4.1* and *VaFld4.2*; PEVs 71.3% and 67.6, respectively) were identified in the 9.65 cM region of chromosome 4 of the F_2_ population affecting flowering and maturation times. Two “first flowering” QTLs with smaller effects were found on chromosome 4. The SNP marker s690-144110 co-segregated with *VaFld4.1*, and the genetic distance between *VaFld4.2* and the SNP marker *s165-116310* was 0.974 cM. The *VaFld4.2* flowering time QTL was present in the same chromosome 4 locus in the F_4_ population, and co-segregated with the s165-116310 SNP marker. Two FLD50 (“time to 50% flowering”) QTLs were located in the scaffold regions of *VaFld4.1* (PEV 73.1%) and *VaFld4.2* (PEV 69.2%), and two other minor flowering time QTLs were located on chromosome 10, with PEVs of 3.9%. One minor QTL was mapped on chromosome 7. PDDM25 (“25% pod maturation”, PEV 55.4%), PDDM50 (“50% pod maturation”, PEV 64.9%), and PDDM75 (“75% pod maturation”, PEV 71.3%) mapped to the same locus *VaFld4.1*, and co-segregated with the s690-144110 SNP marker (Table 4, Figure 2). The remaining two major PDDM75 QTLs, *VaGp4.1* and *VaGp4.2*, mapped to the 2.76 cM region of chromosome 4 (PEVs 47 and 40.9%). The *VaGp4.1* and *VaGp4.2* QTLs for FLD and the *VaGp4.1* QTL for FLD50 were identified in the F_3_ generation.

### Morphology and yield traits

In total, six SD100WT QTLs were detected, four on chromosome 1 and two on chromosome 11 (Figure 3). *VaSd100wt1.1* and *VaSd100wt1.2* were mapped to chromosome 1. *VaSd100wt11.1* located between SNP markers s268-1701932 and s268-1618129 with 0.0420 cM genetic distance on chromosome 11 (Table 4). Three seed-number-per-pod (SDNPPD) QTLs were identified on chromosome 3 (*Snp3.1* PEVs 17.3%) and chromosome 4 (*Snp4.1*, PEV 11.8%). *Snp3.1 was* located at a distance of 0.012 cM between SNP markers s624-5207 and s624-5245, and *Snp3.2* lay on the same mapping region of *Snp3.1*. *Snp4.1* co-segregated with s624-5245 and s80-1776704, respectively. Three QTLs for branch number on the main stem mapped to chromosome 4 (Figure 3, Table 4). *VaBrn4.1* was found on chromosome 4 between SNP markers s690-144110 and s856-165880, and the genetic distance of *VaBrn4.2* was 0.974 cM with a s165-116310 marker (Figure 4). A QTL *VaLfmw2.1* for maximum leaf width (LFMW) co-segregated with a s536-414880 marker on chromosome 2 (PEV 21.2%). A QTL *VaST1-10I9* of stem internode length from the first to the tenth node (ST1I-ST10I) was found on chromosome 9 (PEV 17.3%) and co-segregated with the SNP marker s249-3204596 (Table 4, Figure 4). QTL *VaSdt4.1* of SDT (seed thickness) was mapped on chromosome 4 between SNP markers s58-1489334 and s58-345785, and co-segregated with an S58-1489334 marker (Figure 4, Table 4).

### Qualitative trait gene mapping

**Figure 5:**
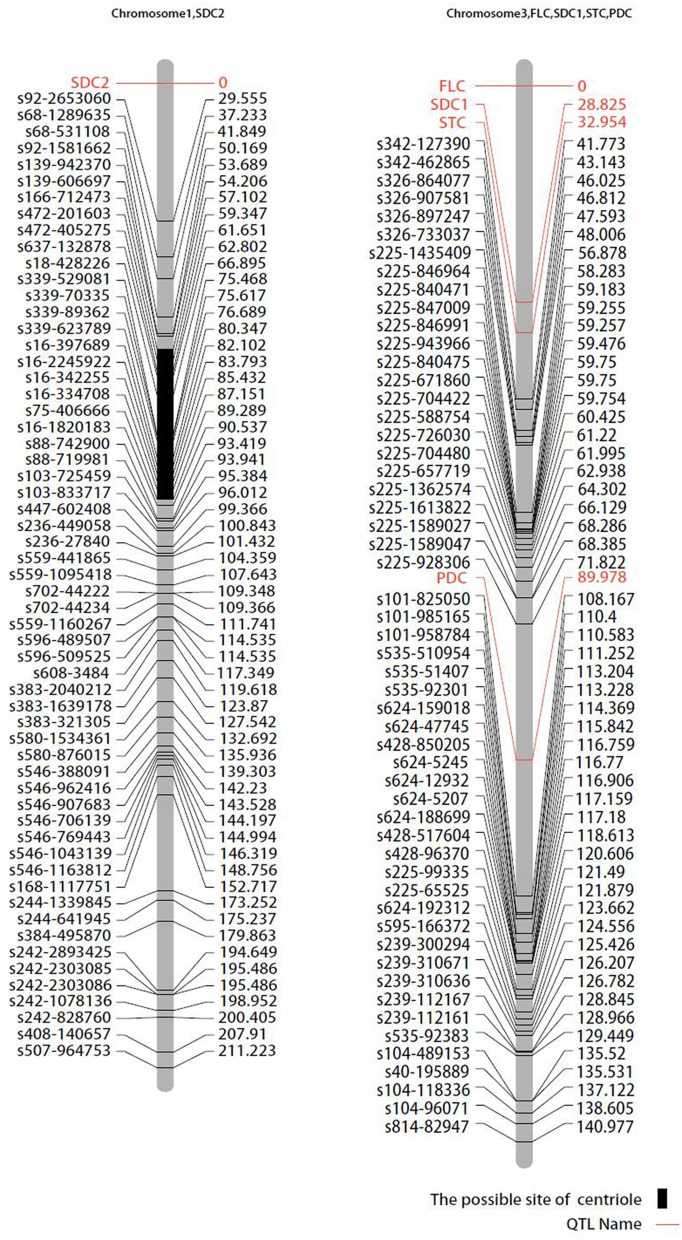
**Mapping of qualitative traits on the map of the population derived from the cross between cultivated and wild adzuki bean**. SDC, seed coat color; FLC, flower color; STC, stem color; PDC, pod color.

Pigmentation-related genes controlling seed coat color (SDC), pod color (PDC), stem color (STC), and flower color (FLC) were mapped to chromosome 3. *VaFcY* controlling yellow flower color mapped to the top of chromosome 3, followed by the black seed coat color gene *VaScB*, and the green stem color gene *VaStcG* (Figure 5). The genetic distances between *VaStcG, VaScB, VaFcY*, and the SNP marker s342-127390 were 8.82, 12.95, and 41.77 cM, respectively. The black pod *VaPcB* gene was located between SNP markers s225-928306 and s101-825050, the genetic distances of which were 18.16 and 19 cM, respectively. The red seed coat color *VaScR* gene mapped on top of chromosome 1.

### Identification of candidate genes

#### Flowering time and pod maturity

Two flowering time candidate genes, *E3-*like phytochrome and transcription factor agamous-like MADS-box loci, and an *AP2* locus were detected in relative mapping regions controlling flowering time, maturity, and seed size.

**Figure 6:**
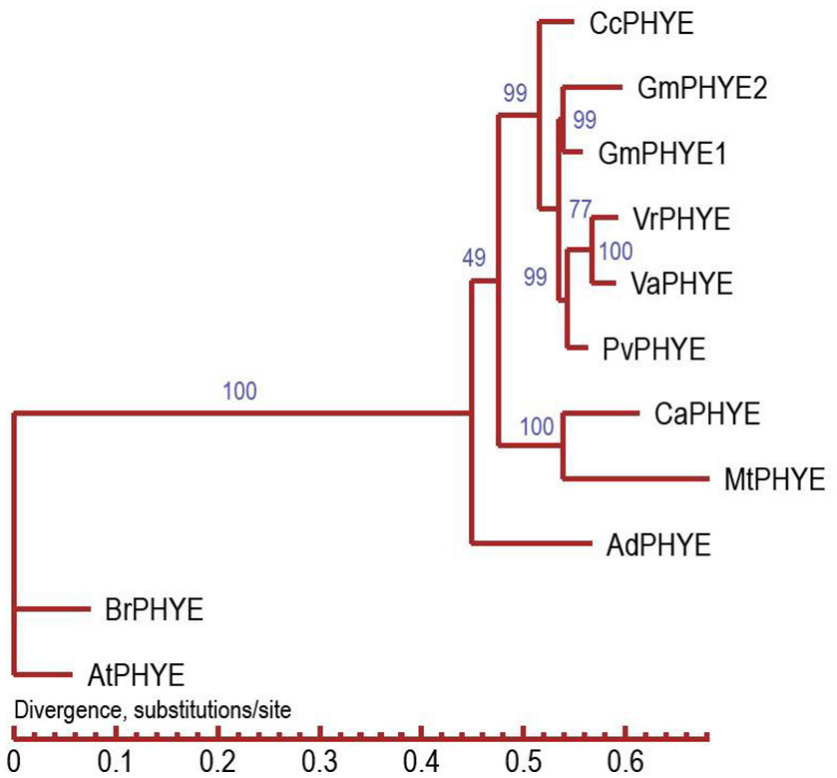
**Phylogenetic tree analysis of adzuki bean PHYE**. CcPHYE (*Cajanus cajan*), GmPHYE1 and GmPHYE2 (*Glycine max*), VrPHYE (*Vigna radiata*), VaPHYE (*Vigna angularis*), PvPHYE (*Phaseolus vulgaris*), CaPHYE (*Cicer arietinum*), MtPHYE (*Medicago truncatula*), AdPHYE (*Arachis duranensis*), BrPHYE (*Brassica rapa*), and AtPHYE (*Arabidopsis thaliana*).

*VaPhyE* (phytochrome E) encodes a protein of 1,121 amino acids. It is located in the interval between 3,182,263 and 3,186,619 bp on chromosome 4. In total, four phytochrome genes—two *VaPhyA*, a *VaPhyB*, and a *VaPhyE* gene—were found in the adzuki bean genome (Table S3). Based on the protein sequence encoded by the *VaPhyE* gene, clustering results showed that adzuki bean *VaPhyE* and mung bean *VrPhyE* have the closest genetic relationship, followed by the common bean *PvPhyE* (Figure 6).

The agamous-like MADS-box is involved in flower development and maturity (de Folter et al., 2005). Two major QTLs controlling flowering time and pod maturity traits (FLD, FLD50, PDDM50, and PDDM100), *VaFld4.1* and *VaFld4.3*, were mapped in the interval from 3,102,255 to 3,616,262 bp on chromosome 4 between SNP markers s690-144110 and s856-165880; s856-165880 was the closest. An agamous-like MADS-box candidate gene, *VaAGL*, encoding a protein of 379 amino acids, was detected in this region. In total, 29 *Agamous*-like MADS-box genes were found in the adzuki bean genome (Table S4).

#### Seed and leaf size

Two *VaAP2/ERF.81* and *VaAP2/ERF.82* candidate genes encoding 278 and 225 amino acids were identified in the SD100WT QTL *VaSd100wt1.2* region of 28,861,763–28,863,078 bp and 29,072,132–29,072,809 bp on chromosome 1, and the closest SNP marker was s168-1117751 (Figure 3). The candidate gene *VaAP2-s4* was found in QTL *VaSdt4.1* of SDT (seed width) from 345,785 to 1,489,334 bp on chromosome 4 between SNP markers s58-345785 and s58-1489334 (Figure 4). *VaAP2-s4* encodes a protein of 208 amino acids. Another candidate gene, *VaAP2/ERF.86*, encoding 638 amino acids, was identified in the mapping region of QTL *VaLfmw2.1* (LFMW, maximum leaf width) from 20,746,593 to 20,747,231 bp on chromosome 2, between SNP markers s536-414880 and s536-414880 Figure 4. In total, 26 *AP2/ERF* genes were identified in the adzuki bean genome (Table S5). *AP2/RAV* genes were absent in adzuki beans, common beans, mung beans, chickpeas, pigeonpeas, and *Medicago truncatula*, but did exist in soybeans.

#### Seed coat color genes

The *VaScB* gene controlling the black seed coat trait (SDC1) was mapped onto the interval from 131,943 to 133,424 bp on chromosome 3 between SNP marker scaffold326-733037 (28.83 cM) and the top of the chromosome 3. A candidate gene, *VaUGT*, was found within this interval. *VaUGT* encodes a protein of UDP flavonoid glycosyl transferase (UGT), with 494 amino acids, associated with flavonoid metabolic pathways. In total, 22 *UGT*-like genes were found in the adzuki bean genome (Table S6, Figure S1).

## Discussion

Gene mapping and QTL detection are very useful for gene cloning, MAS breeding, and trait improvement; however, only a few studies have been performed using SSR markers in adzuki bean (Isemura et al., 2007; Kaga et al., 2008). We identified 26 genomic QTLs associated with agronomic traits and gene loci of four qualitative traits using a high-density SNP genetic map created via RAD sequencing. In this study, F_2_ segregating populations were derived using a wide cross between a cultivated adzuki bean Ass001 (*V. angularis var. angularis*) and a wild adzuki bean CWA108 (*V. angularis* var. *nipponensis*). The seeds of the wild adzuki bean were in dormancy. The seed number of F_2_ individuals was limited. Each of the F_2:3_ lines were planted in two rows in 2014. Each of the F_3:4_ lines were planted in the same manner as the F_2:3_ lines in 2015. From the center of the rows, 10 representational plants per line and 10 plants of each parent were selected to evaluate each trait based on the mean value. Isemura et al. (2007) planted BC_1_F_1:2_ lines and F_2:3_ lines, each consisting of 10 individuals per line in the field, in June 2003 and 2004 at NIAS, and the lines were evaluated based on the mean values for each trait per line and used for QTL mapping. Kaga et al. (2008) used the same planting model and method for QTL identification in adzuki bean. Wu et al. (2013) mapped the QTL of grain shape and size in wheat RILs using 10 representational plants that were selected from the center of two rows to gain phenotypic data. The phenotypic trait data were reliable in this study.

Control of flowering time is critical when pulse crops have to adapt to different ecogeographic environments and photoperiods. In the soybean, a representative warm-season short-day legume, at least 10 *E* loci (*E1*–*E9*) and *J* genes controlling flowering time and maturity have been described (Weller and Ortega, 2015; Cao et al., 2017). In the adzuki bean, a first-flowering-day (FLD) QTL has been described in linkage group 4 (Isemura et al., 2007), and a major relevant QTL (*Fld3.4a.1*, PEV 43.7%) in LG4a; nine FLD and pod maturity QTLs were found to be present on LG2, LG3, LG4a, LG5, LG6a, and LG11 (Kaga et al., 2008). In the present study, we detected 10 QTLs controlling flowering time and pod maturity; the QTLs were on chromosomes 4, 7, and 10. The very relevant *VaFld4.1 and VaFld4.2* loci were on chromosome 4, and the candidate phytochrome E gene *VaPhyE* co-segregated with SNP markers. Phytochrome-A-encoding genes *E3* and *E4* have been identified in the soybean (Liu et al., 2008; Watanabe et al., 2009). *GmE3* and *GmE4* are *phyA*-type (phytochrome A) photoreceptor that plays an important role in the detection of far-red and red light in the soybean, and is associated with early flowering and maturity. *E4*, controls flowering when the LDs are in the low- and far-red regions (Cober and Voldeng, 2001). E3 functions across a wide range of latitudes, whereas allelic differences in E4 are detected only at high latitudes (Lu et al., 2015). GmPHYA1 and GmPHYA2 (E4) may play redundant roles in photomorphogenesis (de-etiolation response and flowering under low R:FR conditions; Liu et al., 2008). Soybean has eight PHY loci including four PHYA, two PHYB, and two PHYE loci. Phylogenetic analysis of soybean suggests that the four paralogous PHY pairs separated (by genomic duplication) at least 13 million years ago, and that the four PHYA copies are remnants of two rounds of such duplication (ca. 58 and 13 million years ago) (Wu et al., 2013). Adzuki bean, like most sequenced legumes, did not experience this *Glycine max*-specific whole genome duplication event (13 million years ago). In this study, we found that there are four PHY loci including two PHYA, a PHYB, and a PHYE loci in adzuki bean, common bean, mung bean, chickpea, pigeonpea, and *Arachis duranensis*, but a PHYA in *Medicago truncatula*. This result confirms that two rounds of whole genome duplication events led to four PHYA and two PHYE copies in soybean. The PHY family includes the A, B, C, D, and E subfamilies; PHYC and PHYD were not found in the adzuki bean, soybean, common bean, chickpea, pigeonpea, mung bean, *Arachis duranensis*, or *M. truncatula* genomes. PHYC and PHYD may have been lost during the evolution of legumes. PHYE was absent in monocotyledon *Oryza sativa* and *Zea mays* (Table S4). *Dt2*, a dominant MADS-domain gene of the *AP1/SQUA* subfamily, has been cloned from the soybean. *Dt2* represses *Dt1* expression in the shoot apical meristem, promoting the early conversion of meristem into reproductive inflorescences; the gene thus promotes semi-determinate stem growth and early flowering (Ping et al., 2014). In this study, 28 *AGAMOUS*-like MADS-box genes were found in the adzuki bean genome for photoperiodic flowering (Table S6). Kim et al. (2014) sequenced a *de novo* assembly of adzuki bean transcript sequences and subjected them to a BLASTP search to identify putative homologs of the 84 *Arabidopsis* genes involved in the circadian clock and photoperiodic flowering pathway. Eleven homologs of the *AGAMOUS*-like MADS-box transcription factor were detected in the adzuki bean. Follow-up studies of candidate gene validation are being planned.

*AP2* genes are representative of the large AP2/EREBP gene family, and are both necessary and sufficient for flower development (Jofuku et al., 1994), stem cell maintenance (Wurschum et al., 2005), seed development, determination of seed size, and seed weight (Jofuku et al., 2005); several *AP2*-encoding proteins repress flowering, redundantly affecting flowering time (Zhu and Helliwell, 2011). Four *VaAP2* were identified in QTL mapping intervals of seed weight, leaf size, and seed thickness in adzuki bean. We concluded that these *VaAP2* might function in regulating organ size, but their function still requires validation. If leaf width candidate gene *VaAP2-l2* is involved in or regulates seed size, this should be analyzed in future studies.

The black seed coat color *VaScB* and red seed coat color *VaScR* genes mapped to chromosomes 3 and 1, respectively. Kaga et al. (2008) mapped the red seed coat locus near the SSR marker CEDG053 on linkage group 1 (LG1). Genes that control red or tan seed coat colors, and black seed mottling have been mapped to linkage groups 1 and 4, respectively (Isemura et al., 2007). A minor QTL *OLB2* explaining 6% of the total variance in redness is located on LG1 (Horiuchi et al., 2015). *VaScR* will be finely mapped by reference to the closest SNP and SSR markers in follow-up studies. *UGT* is a member of the flavonoid metabolic pathway, and is involved in anthocyanin biosynthesis. The enzymes O-methyltransferase, rhamnosyl transferase, and UDP flavonoid glucosyl transferase together synthesize anthocyanins from 3-OH-anthocyanidins. UDP glycosyl transferase is essential for anthocyanin synthesis. The enzyme may be involved in delphinidin synthesis; this pigment is blue–black in color, and may be responsible for the difference between black (pigment present) and brown (pigment absent) seed coat colors (Lepiniec et al., 2006). We designed primers and found a new SNP cosegregative marker with *VaScB*. The proposed function of *VaUGT* for synthesis of a black seed coat needs to be verified experimentally. A *UGT78K1* (UDP-glucose: flavonoid-3-O-glycosyltransferase) gene, imparting a black seed coat, has been isolated from the black (iRT) soybean (Kovinich et al., 2010). *VaUGT* belongs to 73C3-like UDP-glycosyl transferase, and is different from *GmUGT78K1*; its function will need to be further verified.

## Author contributions

PW designed and managed the project and wrote the manuscript. YL prepared DNA samples for RAD sequencing, and engaged in data investigation and analysis. YL, KY, WY, and JJ performed QTL analyses. YL and KY identified candidate flowering time genes. BZ, YSL, and TW participated in trait investigations, data analysis, and field experiments. LC and ZY mapped qualitative traits and identified relevant candidate genes. CC analyzed the phylogenetic tree. KY contributed helpful suggestions.

### Conflict of interest statement

The authors declare that the research was conducted in the absence of any commercial or financial relationships that could be construed as a potential conflict of interest.

## Supplementary material

Table S1 **Phenotype Data of Parents and F_**2**_ , F_**3**_ and F_**4**_ Populations**.

Table S2 **Comparison of sequences assembly between parents and 150 individuals of F_**2**_ by RAD-sequencing**.

Table S3 **Phytochrome genes in the adzuki bean and comparison to sequenced legume and other plant genomes**.

Table S4 *****Agamous-like MADS-box*** genes in the adzuki bean and comparison to sequenced legume and other plant genomes**.

Table S5 *****AP2/EREBF*** genes in the adzuki bean and comparison to sequenced legume and other plant genomes**.

Table S6 **UDP flavonoid glycosyl transferase (UGT) genes in the adzuki bean and comparison to sequenced legume and other plant genomes**.

Figure S1 **Phylogenetic tree analysis of adzuki bean ***UGT***-like genes**.
