# Supplementary material for: Identification of QTL and Qualitative Trait Loci for Agronomic Traits Using SNP Markers in the Adzuki Bean
Source: Front Plant Sci. 2017 May 19;8:840. doi: 10.3389/fpls.2017.00840 (PMC5437206; doi:10.3389/fpls.2017.00840)
Supplement: Table S2 — Comparison of sequences assembly between parents and 150 individuals of F2 by RAD-sequencing. [file Table2-5.DOCX]

Supplementary Table S2 Comparison of sequences assembly between parents and 150 individuals of F_2_ by RAD-sequencing

| Sample | Total_reads_No. | Map_readsNo. | Map_ratio | Uniq_map_reads_No. | Uniq_map_ratio |
| --- | --- | --- | --- | --- | --- |
| AS002 | 10000710 | 9558619 | 0.96 | 7261400 | 0.73 |
| AS003 | 9490786 | 9082325 | 0.96 | 6918037 | 0.73 |
| AS004 | 9468292 | 9052731 | 0.96 | 6876645 | 0.73 |
| AS005 | 7983378 | 7652938 | 0.96 | 5874047 | 0.74 |
| AS006 | 8268126 | 7912038 | 0.96 | 5949877 | 0.72 |
| AS007 | 8975522 | 8567417 | 0.95 | 6467022 | 0.72 |
| AS008 | 8932498 | 8510951 | 0.95 | 6433906 | 0.72 |
| AS009 | 11511540 | 11016334 | 0.96 | 8241567 | 0.72 |
| AS010 | 10403132 | 9952566 | 0.96 | 7358447 | 0.71 |
| AS011 | 8715676 | 8345551 | 0.96 | 6029893 | 0.69 |
| AS012 | 11241524 | 10741576 | 0.96 | 7935303 | 0.71 |
| AS013 | 8651518 | 8254433 | 0.95 | 6233719 | 0.72 |
| AS014 | 6807244 | 6522391 | 0.96 | 4932354 | 0.72 |
| AS015 | 6276964 | 6060284 | 0.97 | 4582509 | 0.73 |
| AS016 | 9026762 | 8666504 | 0.96 | 6499413 | 0.72 |
| AS017 | 9806152 | 9383601 | 0.96 | 7103835 | 0.72 |
| AS018 | 9008614 | 8628999 | 0.96 | 6644811 | 0.74 |
| AS019 | 10834218 | 10374371 | 0.96 | 7981929 | 0.74 |
| AS020 | 10786766 | 10365415 | 0.96 | 7918795 | 0.73 |
| AS021 | 9237178 | 8834412 | 0.96 | 6825727 | 0.74 |
| AS022 | 10692188 | 10249450 | 0.96 | 7679543 | 0.72 |
| AS023 | 8824944 | 8430433 | 0.96 | 6356449 | 0.72 |
| AS024 | 10787836 | 10328276 | 0.96 | 7697307 | 0.71 |
| AS025 | 11281310 | 10681042 | 0.95 | 7906829 | 0.7 |
| AS026 | 8284594 | 7991510 | 0.96 | 6190354 | 0.75 |
| AS028 | 8301656 | 7955655 | 0.96 | 5972454 | 0.72 |
| AS029 | 10639298 | 10229693 | 0.96 | 7787438 | 0.73 |
| AS030 | 10786814 | 10344539 | 0.96 | 7823793 | 0.73 |
| AS031 | 9635748 | 9279238 | 0.96 | 6886892 | 0.71 |
| AS032 | 8156338 | 7816313 | 0.96 | 5904533 | 0.72 |
| AS033 | 9715780 | 9311003 | 0.96 | 6884687 | 0.71 |
| AS035 | 9468944 | 9033367 | 0.95 | 6720626 | 0.71 |
| AS036 | 11151978 | 10681741 | 0.96 | 7957934 | 0.71 |
| AS037 | 6967604 | 6712316 | 0.96 | 5079615 | 0.73 |
| AS038 | 10240478 | 9814773 | 0.96 | 7301249 | 0.71 |
| AS039 | 9260342 | 8922604 | 0.96 | 6848283 | 0.74 |
| AS040 | 9442212 | 8951093 | 0.95 | 6606620 | 0.7 |
| AS041 | 8195332 | 7836571 | 0.96 | 5851726 | 0.71 |
| AS042 | 9458172 | 9096288 | 0.96 | 6971148 | 0.74 |
| AS043 | 5959340 | 5748615 | 0.96 | 4311452 | 0.72 |
| AS044 | 8581864 | 8265345 | 0.96 | 6293121 | 0.73 |
| AS045 | 7500936 | 7239769 | 0.97 | 5440735 | 0.73 |
| AS046 | 9234828 | 8865208 | 0.96 | 6717925 | 0.73 |
| AS047 | 8864302 | 8484487 | 0.96 | 6483768 | 0.73 |
| AS048 | 9235182 | 8838928 | 0.96 | 6514575 | 0.71 |
| AS049 | 4038372 | 3806612 | 0.94 | 2836189 | 0.7 |
| AS050 | 7325550 | 7083380 | 0.97 | 5394427 | 0.74 |
| AS051 | 6435348 | 6209209 | 0.96 | 4816897 | 0.75 |
| AS052 | 8935368 | 8558528 | 0.96 | 6505237 | 0.73 |
| AS053 | 9607406 | 9225501 | 0.96 | 7108017 | 0.74 |
| AS054 | 9630232 | 9215411 | 0.96 | 7049168 | 0.73 |
| AS055 | 8829220 | 8444944 | 0.96 | 6222350 | 0.7 |
| AS056 | 7433520 | 7121917 | 0.96 | 5383123 | 0.72 |
| AS057 | 7306314 | 7058713 | 0.97 | 5147840 | 0.7 |
| AS058 | 8689630 | 8334375 | 0.96 | 6411884 | 0.74 |
| AS059 | 11776320 | 11279460 | 0.96 | 8747894 | 0.74 |
| AS060 | 10609354 | 10141444 | 0.96 | 7630898 | 0.72 |
| AS061 | 9979908 | 9593104 | 0.96 | 7222734 | 0.72 |
| AS062 | 9047032 | 8681918 | 0.96 | 6595895 | 0.73 |
| AS063 | 10940692 | 10479172 | 0.96 | 7847903 | 0.72 |
| AS064 | 8630236 | 8275362 | 0.96 | 6317646 | 0.73 |
| AS065 | 625266 | 606384 | 0.97 | 458636 | 0.73 |
| AS066 | 10511016 | 10122468 | 0.96 | 7628017 | 0.73 |
| AS067 | 8889390 | 8580401 | 0.97 | 6490994 | 0.73 |
| AS068 | 10593636 | 10174539 | 0.96 | 7666729 | 0.72 |
| AS069 | 8684130 | 8363890 | 0.96 | 6415248 | 0.74 |
| AS070 | 6341524 | 6129202 | 0.97 | 4605589 | 0.73 |
| AS071 | 8720612 | 8388364 | 0.96 | 6308936 | 0.72 |
| AS072 | 10672714 | 10261683 | 0.96 | 7698609 | 0.72 |
| AS073 | 10313546 | 9890433 | 0.96 | 7489307 | 0.73 |
| AS074 | 8269316 | 7939173 | 0.96 | 5885635 | 0.71 |
| AS075 | 8660678 | 8321039 | 0.96 | 6304085 | 0.73 |
| AS076 | 8480930 | 8172281 | 0.96 | 6177092 | 0.73 |
| AS077 | 7783578 | 7510371 | 0.96 | 5713594 | 0.73 |
| AS078 | 7687584 | 7396516 | 0.96 | 5677201 | 0.74 |
| AS079 | 10142318 | 9738017 | 0.96 | 7509227 | 0.74 |
| AS080 | 17252012 | 16539847 | 0.96 | 12796788 | 0.74 |
| AS081 | 14909506 | 14296077 | 0.96 | 10780692 | 0.72 |
| AS082 | 10464036 | 10051375 | 0.96 | 7724103 | 0.74 |
| AS083 | 14391484 | 13851517 | 0.96 | 10753551 | 0.75 |
| AS084 | 15255966 | 14661209 | 0.96 | 11267182 | 0.74 |
| AS085 | 15228284 | 14593317 | 0.96 | 11142680 | 0.73 |
| AS086 | 19043668 | 18246837 | 0.96 | 14187513 | 0.74 |
| AS088 | 16965302 | 16318825 | 0.96 | 12704151 | 0.75 |
| AS089 | 8551546 | 8197187 | 0.96 | 6084334 | 0.71 |
| AS090 | 16494210 | 15824793 | 0.96 | 12162623 | 0.74 |
| AS091 | 10287802 | 9893773 | 0.96 | 7468152 | 0.73 |
| AS092 | 5777348 | 5562198 | 0.96 | 4280987 | 0.74 |
| AS093 | 13622950 | 13059600 | 0.96 | 10007260 | 0.73 |
| AS094 | 13894440 | 13325564 | 0.96 | 10034577 | 0.72 |
| AS095 | 14754764 | 14192166 | 0.96 | 11085221 | 0.75 |
| AS096 | 14948752 | 14371231 | 0.96 | 11191710 | 0.75 |
| AS097 | 15195828 | 14611014 | 0.96 | 10999453 | 0.72 |
| AS098 | 14885640 | 14260813 | 0.96 | 10839583 | 0.73 |
| AS099 | 16708722 | 16047619 | 0.96 | 12284989 | 0.74 |
| AS100 | 9365754 | 9026296 | 0.96 | 6688649 | 0.71 |
| AS101 | 13566452 | 12995503 | 0.96 | 9893901 | 0.73 |
| AS102 | 16485338 | 15808756 | 0.96 | 12189306 | 0.74 |
| AS103 | 10210434 | 9801389 | 0.96 | 7472861 | 0.73 |
| AS104 | 9020656 | 8642178 | 0.96 | 6649274 | 0.74 |
| AS105 | 8291508 | 7940003 | 0.96 | 6091813 | 0.73 |
| AS106 | 10685532 | 10256025 | 0.96 | 7866606 | 0.74 |
| AS107 | 11578954 | 11089210 | 0.96 | 8431493 | 0.73 |
| AS109 | 10336760 | 9917792 | 0.96 | 7687169 | 0.74 |
| AS110 | 11608140 | 11131555 | 0.96 | 8489893 | 0.73 |
| AS111 | 10448588 | 10035947 | 0.96 | 7633772 | 0.73 |
| AS112 | 7901552 | 7623729 | 0.96 | 5794792 | 0.73 |
| AS113 | 11138390 | 10732924 | 0.96 | 8182478 | 0.73 |
| AS114 | 9899376 | 9489959 | 0.96 | 7186420 | 0.73 |
| AS115 | 11549470 | 11044695 | 0.96 | 8274412 | 0.72 |
| AS116 | 7988900 | 7646009 | 0.96 | 5821827 | 0.73 |
| AS117 | 9980432 | 9567101 | 0.96 | 7091724 | 0.71 |
| AS118 | 12559730 | 12022780 | 0.96 | 9090825 | 0.72 |
| AS119 | 10074196 | 9672866 | 0.96 | 7239797 | 0.72 |
| AS120 | 9064922 | 8710638 | 0.96 | 6185081 | 0.68 |
| AS121 | 10710424 | 10255583 | 0.96 | 7798256 | 0.73 |
| AS122 | 9471998 | 9132967 | 0.96 | 6922934 | 0.73 |
| AS123 | 9229738 | 8889217 | 0.96 | 6860231 | 0.74 |
| AS125 | 7631764 | 7329983 | 0.96 | 5522826 | 0.72 |
| AS126 | 8683538 | 8327296 | 0.96 | 6251988 | 0.72 |
| AS127 | 10074650 | 9707186 | 0.96 | 7436916 | 0.74 |
| AS128 | 9751952 | 9351241 | 0.96 | 7151934 | 0.73 |
| AS129 | 6094764 | 5889688 | 0.97 | 4412706 | 0.72 |
| AS130 | 8038806 | 7744319 | 0.96 | 5813640 | 0.72 |
| AS131 | 5199408 | 5023306 | 0.97 | 3863391 | 0.74 |
| AS132 | 10438210 | 10013532 | 0.96 | 7581050 | 0.73 |
| AS133 | 11016418 | 10528969 | 0.96 | 7893146 | 0.72 |
| AS134 | 8986664 | 8618371 | 0.96 | 6621926 | 0.74 |
| AS135 | 8845462 | 8530831 | 0.96 | 6430721 | 0.73 |
| AS136 | 10259802 | 9834085 | 0.96 | 7349968 | 0.72 |
| AS137 | 10248300 | 9872862 | 0.96 | 7535894 | 0.74 |
| AS138 | 8701240 | 8342317 | 0.96 | 6469515 | 0.74 |
| AS139 | 10313094 | 9906973 | 0.96 | 7672152 | 0.74 |
| AS140 | 8188998 | 7799489 | 0.95 | 5834809 | 0.71 |
| AS141 | 8819838 | 8483693 | 0.96 | 6525507 | 0.74 |
| AS142 | 9538008 | 9226779 | 0.97 | 7106952 | 0.75 |
| AS143 | 8008730 | 7633333 | 0.95 | 5844949 | 0.73 |
| AS144 | 8912312 | 8557468 | 0.96 | 6370597 | 0.71 |
| AS145 | 12845586 | 12271482 | 0.96 | 9292280 | 0.72 |
| AS146 | 12374604 | 11823478 | 0.96 | 8894233 | 0.72 |
| AS147 | 14811442 | 14189303 | 0.96 | 10673073 | 0.72 |
| AS148 | 12463486 | 11933370 | 0.96 | 9140366 | 0.73 |
| AS149 | 12433862 | 11933027 | 0.96 | 9325684 | 0.75 |
| AS150 | 15252694 | 14636755 | 0.96 | 11369801 | 0.75 |
| AS156 | 12744854 | 12209805 | 0.96 | 9249954 | 0.73 |
| AS159 | 13345956 | 12855576 | 0.96 | 9794355 | 0.73 |
| AS163 | 20484434 | 19639480 | 0.96 | 14785426 | 0.72 |
| AS172 | 19257750 | 18492385 | 0.96 | 14340310 | 0.74 |
| AS184 | 7386428 | 7103882 | 0.96 | 5517648 | 0.75 |
| AS188 | 15332766 | 14754571 | 0.96 | 11221271 | 0.73 |
| ass001 | 8721882 | 8388058 | 0.96 | 6418606 | 0.74 |
| CWA108 | 10355818 | 9886992 | 0.95 | 7516722 | 0.73 |

Supplementary Table S3 Phytochrome genes in the adzuki bean and comparison to

sequenced legume and other plant genomes

| Species | Total | PHYA | PHYB | PHYC | PHYD | PHYE |
| --- | --- | --- | --- | --- | --- | --- |
| *Vigna angularis* | 4 | 2 (chromosome 2,3) | 1(chromosome 9) | 0 | 0 | 1(chromosome 4) |
| *Arachis duranensis* | 4 | 2 | 1 | 0 | 0 | 1 |
| *Glycine max* | 8 | 4 | 2 | 0 | 0 | 2 |
| *Cicer arietinum* | 4 | 2 | 1 | 0 | 0 | 1 |
| *Medicago truncatula* | 3 | 1 | 1 | 0 | 0 | 1 |
| *Cajanus cajan* | 4 | 2 | 1 | 0 | 0 | 1 |
| *Phaseolus vulgaris* | 4 | 2 | 1 | 0 | 0 | 1 |
| *Vigna radiata* | 4 | 2 | 1 | 0 | 0 | 1 |
| *Arabidopsis thaliana* | 5 | 1 | 1 | 1 | 1 | 1 |
| *Brassica rapa* | 5 | 2 | 1 | 1 | 0 | 1 |
| *Oryza sativa* | 3 | 1 | 1 | 1 | 0 | 0 |
| *Zea mays* | 6 | 2 | 2 | 2 | 0 | 0 |

Supplementary Table S4  *Agamous-like MADS-box* genes in the adzuki bean and comparison to sequenced legume and other plant genomes

| Species | Total | MADS/AGL | MADS/TF | MADS protein |
| --- | --- | --- | --- | --- |
| *Vigna angularis* | 71 | 29 | 1 | 41 |
| *Arabidopsis thaliana* | 116 | 65 | 26 | 25 |
| *Brassica rapa* | 159 | 86 | 22 | 51 |
| *Cicer arietinum* | 64 | 20 | 6 | 38 |
| *Glycine max* | 164 | 43 | 6 | 115 |
| *Oryza sativa* | 59 | 13 | 0 | 46 |
| *Medicago truncatula* | 116 | 67 | 9 | 40 |
| *Cajanus cajan* | 77 | 25 | 3 | 49 |
| *Phaseolus vulgaris* | 66 | 21 | 2 | 43 |
| *Vigna radiata* | 72 | 25 | 1 | 46 |
| *Zea mays* | *85* | *12* | *0* | *73* |

Supplementary Table S5 *AP2/EREBF* genes in the adzuki bean and comparison to sequenced legume and other plant genomes

| AP2/EREBF |  | RAV | | EREBF | | | | | AP2 |
| --- | --- | --- | --- | --- | --- | --- | --- | --- | --- |
|  | Total | RAV | RAV1 | RAV2 | DREB/RAP | ERF | ERE/DRE | TINY | AP2 |
| *Vigna angularis* | 118 | 0 | 0 | 0 | 14 | 26 | 39 | 6 | 33 |
| *Arabidopsis thaliana* | 165 | 2 | 1 | 1 | 15 | 17 | 40 | 9 | 80 |
| *Brassica rapa* | 295 | 3 | 2 | 1 | 32 | 38 | 72 | 18 | 129 |
| *Cicer arietinum* | 94 | 0 | 0 | 0 | 15 | 26 | 28 | 5 | 20 |
| *Glycine max* | 219 | 2 | 0 | 2 | 28 | 50 | 69 | 9 | 59 |
| *Oryza sativa* | 61 | 4 | 1 | 3 | 2 | 8 | 19 | 4 | 20 |
| *Medicago truncatula* | 126 | 0 | 0 | 0 | 19 | 30 | 49 | 2 | 26 |
| *Cajanus cajan* | 121 | 0 | 0 | 0 | 17 | 30 | 42 | 4 | 28 |
| *Phaseolus vulgaris* | 116 | 0 | 0 | 0 | 14 | 29 | 33 | 8 | 32 |
| *Vigna radiata* | 121 | 0 | 0 | 0 | 9 | 29 | 40 | 7 | 36 |
| *Zea mays* | 91 | 4 | 0 | 4 | 10 | 13 | 23 | 3 | 34 |
