# Supplementary figures and images for: Identification of QTL and Qualitative Trait Loci for Agronomic Traits Using SNP Markers in the Adzuki Bean
Source: Front Plant Sci. 2017 May 19;8:840. doi: 10.3389/fpls.2017.00840 (PMC5437206; doi:10.3389/fpls.2017.00840)

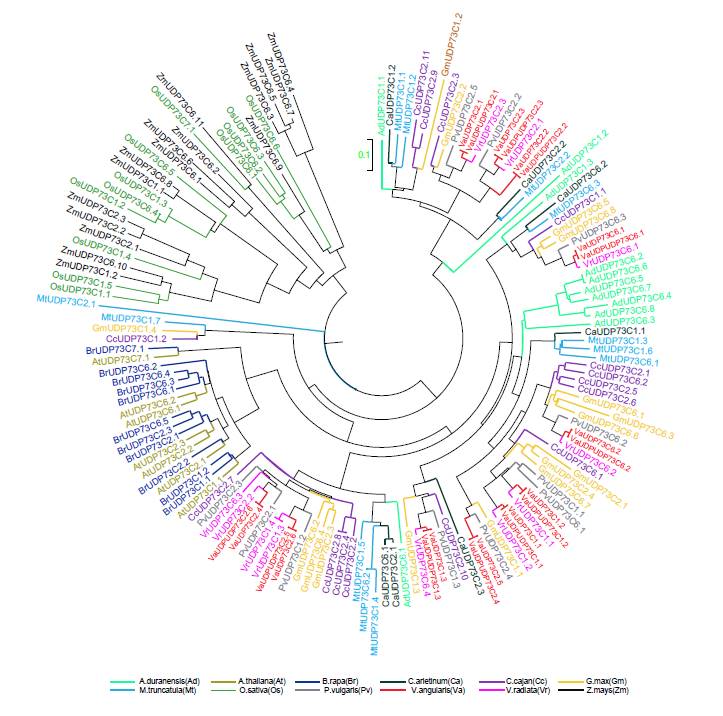

Supplement: Figure S1 — Phylogenetic tree analysis of adzuki bean UGT-like genes. [file Image1.JPEG]
